# Telomere Length and Male Fertility

**DOI:** 10.3390/ijms22083959

**Published:** 2021-04-12

**Authors:** Manuel Gentiluomo, Alice Luddi, Annapaola Cingolani, Marco Fornili, Laura Governini, Ersilia Lucenteforte, Laura Baglietto, Paola Piomboni, Daniele Campa

**Affiliations:** 1 Department of Biology, University of Pisa, 56126 Pisa, Italy; manuel.gentiluomo@biologia.unipi.it (M.G.); annacingolani96@gmail.com (A.C.); daniele.campa@unipi.it (D.C.); 2 Department of Molecular and Developmental Medicine, Siena University, 53100 Siena, Italy; alice.luddi@unisi.it (A.L.); laura.governini@unisi.it (L.G.); 3 Department of Clinical and Experimental Medicine, University of Pisa, 56126 Pisa, Italy; marco.fornili@med.unipi.it (M.F.); ersilia.lucenteforte@unipi.it (E.L.); laura.baglietto@unipi.it (L.B.)

**Keywords:** telomere length, spermatogenesis, infertility, STL, LTL1

## Abstract

Over the past decade, telomeres have attracted increasing attention due to the role they play in human fertility. However, conflicting results have been reported on the possible association between sperm telomere length (STL) and leukocyte telomere length (LTL) and the quality of the sperm parameters. The aim of this study was to run a comprehensive study to investigate the role of STL and LTL in male spermatogenesis and infertility. Moreover, the association between the sperm parameters and 11 candidate single nucleotide polymorphisms (SNPs), identified in the literature for their association with telomere length (TL), was investigated. We observed no associations between sperm parameters and STL nor LTL. For the individual SNPs, we observed five statistically significant associations with sperm parameters: considering a *p* < 0.05. Namely, *ACYP2*˗rs11125529 and decreased sperm motility (*p* = 0.03); *PXK*˗rs6772228 with a lower sperm count (*p* = 0.02); *NAF1*˗rs7675998 with increased probability of having abnormal acrosomes (*p* = 0.03) and abnormal flagellum (*p* = 0.04); *ZNF208*˗rs8105767 and reduction of sperms with normal heads (*p* = 0.009). This study suggests a moderate involvement of telomere length in male fertility; however, in our analyses four SNPs were weakly associated with sperm variables, suggesting the SNPs to be pleiotropic and involved in other regulatory mechanisms independent of telomere homeostasis, but involved in the spermatogenic process.

## 1. Introduction

The World Health Organization (WHO) defines infertility as the inability of a couple to conceive naturally after one year of regular unprotected sexual intercourse [1]. Male infertility is involved in 20–70% of infertile couples [2,3,4] and it is a complex multifactorial pathological condition with profoundly different phenotypic presentations, from a complete absence of spermatozoa in semen (azoospermia) to various alterations of sperm quality [3,5]. Surprisingly, in about 40% of the males with impaired spermatogenesis the aetiology remains unknown after a complete diagnostic workup [5], indicating the need for a better understanding of the biology of the condition and its aetiology. There are several known lifestyle factors associated with male infertility, including body mass index (BMI), tobacco consumption, alcohol abuse, and peripheral vascular disease [6,7,8,9]. Several molecular and epidemiologic studies point towards a decisive role of genetics in male infertility. Structural chromosome aberrations, especially of the Y chromosome, as well as aberration and karyotype alteration, such as Klinefelter syndrome, have been frequently reported in infertile males [10,11,12,13,14]. Single nucleotide polymorphisms (SNPs) have also been investigated in relation to male infertility: two genome-wide association studies (GWAS) conducted in Caucasians suggested several potentially interesting associations, but with none reaching genome-wide significance [15,16]. Two GWAS conducted in the Chinese population identified five loci significant at a genome-wide level. Moreover, Kosova and colleagues carried out a small GWAS in the Hutterites and proposed nine more suggestive (*p* ≤ 10^−4^) susceptibility SNPs [17]. Finally, Sato and colleagues in a GWAS conducted in the Japanese population reported an additional risk variant [18]. In addition to GWAS, several gene candidate studies have been conducted analyzing infertility parameters as outcomes [19,20,21,22,23,24]. Considering that male infertility is a common condition and almost half of the cases are idiopathic, the discovery of novel genetic variants is of the utmost importance to further our knowledge on this field of research.

Another layer of complexity in the aetiology of male infertility is due to the role of telomeres [25,26,27,28,29,30,31], with several studies investigating sperm telomere length (STL) and leukocyte telomere length (LTL) in male infertility [26,29,31,32,33,34,35]. These studies have been conducted using different study designs, measuring techniques, exposure variables and outcomes, and have a limited sample size, ranging from 20 to 100 in Caucasian males. These studies report contrasting results, with some authors reporting a positive association between longer STL or LTL and better quality of the sperm parameters, some authors reporting the opposite, and others reporting no association at all [28,36]. Various techniques, such as real-time polymerase chain reaction (RT-PCR), quantitative fluorescent in situ hybridization (Q-FISH), single telomere length analysis (STELA), terminal restriction fragment (TRF), and primed in situ subtype of Q-FISH (PRINS) were used to measure STL/LTL [37,38,39]. Differences in techniques, sample storage, study settings, and severity of the diagnosis contribute to the heterogeneity of the results, making it difficult to identify the relation between telomere length and infertility in men. A possible approach is suggested by the observation that LTL variability is under genetic control, and GWAS have identified 11 SNPs that predict LTL and therefore have been used as a surrogate for the direct measure of LTL and to evaluate the causative association between LTL and the risk of several human disease in large epidemiologic studies [40,41,42,43,44,45,46,47]. We have called teloscore the score based on genetic determinants of LTL, as previously reported [47,48]. With these premises, our objective was to run a comprehensive study to investigate the role of STL and LTL in male infertility. We used two complementary strategies, analyzing STL via standard quantitative PCR and investigating LTL using the 11 SNPs identified in the literature singularly or in combination (teloscore) in up to 599 male patients of Caucasian origin. The secondary goal of this project was to validate the teloscore as a proxy of telomere length in sperm cells, an attempt that has never been made so far.

## 2. Results

### 2.1. Study Subjects Included in the Analyses

The age and the mean sperm parameters of the enrolled patients are summarized in Table 1.

Seminal parameters were ranked into four categories according to WHO 2010 percentiles. The A category refers to value lower than 5th percentile, the B category to value ranging from 5th to 50th percentiles, the C category to value ranging from 50th to 95th percentiles, and the D category to value higher than the 95th percentile. Based on these four sperm parameters, we computed the “ABCD score” to reduce the number of parameters describing the sperm quality.

After genotyping quality control out of 599 subjects, 14 were discarded because they had a call rate lower than 80% (~9 SNPs of 11). A subset (494) of the study subjects had 100% SNP call rate. To compute comparable score values for all study subjects, we considered a scaled score for each of 585 subjects with a call-rate >80% to obtain scaled unweighted and weighted scores. The relative STL was measured in 239 DNA sperm samples because the remaining study subjects did not have sufficient sperm DNA.

### 2.2. Association between STL and Sperm Parameters

We observed an inverse association between age and sperm quality (*p* < 0.05) (Table 2).

No statistically significant association was observed between STL from RT-PCR and sperm parameters, neither using Pfaffl as a continuous variable nor dividing it in quintiles. The Pfaffl method is a widely used mathematical model to quantify the relative ratio of target sequence (STL) in comparison to a reference gene (ALB) [49,50]. The only exception was a significantly lower percentage of sperm cells with a normal morphology for the fourth vs. the first quintile of STL: coeff. −2.25 (CI95% −4.40–−0.11; *p* = 0.04). The results of this analysis are shown in Table 3.

### 2.3. Association between Teloscore and Sperm Parameters

We observed a reduction of the spermatozoa with normal acrosome (*p* = 0.04) comparing the fifth quintile versus the first quintile of the unweighted teloscore. For the weighted teloscore we observed a reduction of the spermatozoa with normal head (*p* = 0.04) comparing the second quintile versus the first quintile. The other analyses did not show any statistically significant associations (Table S1).

### 2.4. Association between SNPs, Teloscore, and STL

We observed no statistically significant association between the teloscore and the telomere length measured in sperm cells, however when analyzing the association of the single SNPs used to compose the teloscore and the STL, we observed that the C allele of *TERC*˗rs10936599 and the A allele of *TERT*˗rs2736100 were both associated with longer sperm telomere (*p* = 0.040 and *p* = 0.049, respectively). These results are shown in Table 4.

### 2.5. Association between Individual SNPs and Sperm Parameters

**Figure 1:**
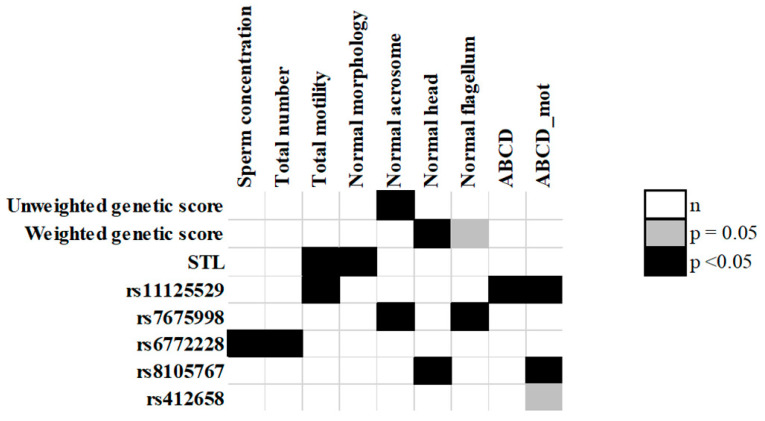
Heatmap of the results of association analyses between sperm parameters and telomere length. Analyses performed using a generalized linear model adjusted by age and smoke status, including the reaction plate as randomic effect variable. In black the association that reported in at least one analysis model a *p* value < 0.05, in dark grey the suggestive associations with a *p* value equal 0.05 in at least one analysis model.

We tested the association of the selected SNPs with the sperm parameters, and we observed 5 statistically significant associations. Namely, the *ACYP2*˗rs11125529˗C allele was associated with decreased sperm motility (*p* = 0.03); the *PXK*˗rs6772228-A allele with a lower sperm count (*p* = 0.02); the *NAF1˗*rs7675998˗A allele with increased probability of having abnormal acrosomes (*p* = 0.03) and abnormal flagellum (*p* = 0.04). Finally, the strongest association we observed was between the *ZNF208*˗rs8105767˗G allele and reduction of sperms with normal heads (*p* = 0.009). The results of the association showing a *p*-value <0.05 are reported in Table 5, Figure 1, while the results of all the analyses are shown in Table S2. After applying correction for multiple testing none of the association remained statistically significant.

### 2.6. Sperm ABCD Scores Association Analysis

An association was observed between the ABCD score and the ABCD_mot score and the *ACYP2*˗rs11125529 SNP: carriers of the C allele had an increased chance of being in the lower end of both scores, corresponding to an overall worse quality of the sperm parameters. *ZNF208*˗rs8105767˗G allele carriers were associated with worse motility. The results of these analyses are shown in Table 6. STL analysis did not show any statistically significant association with ABCD score and ABCD_mot (Table 3).

### 2.7. Functional Effects of the SNPs

For the SNPs that showed an association in the analyses, we searched a possible biological explanation through bioinformatics tools. RegulomeDB assigned a score of 1f to ZNF208˗rs8105767, suggesting a high possibility of being functional through the regulation of gene expression. In addition, RegulomeDB gives a score of 3a to TERC˗rs10936599 indicating a moderate chance for the SNP to have a functional role. GTEx showed an eQTL and/or sQTL in the testis for *ACYP2*˗rs11125529, PXK˗rs6772228, *TERC*˗rs10936599, and *ZNF208*˗rs8105767. Table 7 shows the relevant SNPs with their functional annotation.

## 3. Discussion

In this large sample of male patients of Caucasian origin, we observed no associations between sperm parameters and STL nor LTL. However, among the sperm parameters, three of them (sperm motility, sperm count, sperm morphology) were associated with 4 of the 11 candidate SNPs identified in the literature for being genetic proxies of telomere length.

Telomere length in mature spermatozoa is longer compared to all other human cells, due to the delayed switching off of the telomerase activity in order to deliver intact telomeres to the future progeny, highlighting the crucial importance of telomeres in male gametes. In our study, we investigated the association between telomere length and sperm parameters in one of the largest studies on male infertility, applying two different strategies, the direct measure of STL through RT-PCR and an indirect measure based on the teloscore. The latter approach was never attempted so far, in male infertility, however it has been successfully used in many human diseases as a proxy of telomere length [40,41,42,43,44,45,46,47].

The RT-PCR analysis showed that there was no association between STL and sperm parameters. These results are in agreement with Turner and Thilagavathi’s studies [32,33]. However, several studies have reported an association between both longer and shorter telomere length with various sperm parameters. The studies conducted on Caucasian are generally very small with an average sample size of 40 individuals as reviewed by Vasilopoulos and colleagues, and very heterogenous in terms of study design (e.g., age of subjects enrolled, sample size,) outcome (severity of infertility), and results [28,36].

Our results obtained with the teloscore are in agreement with what we found using the qPCR (Pfaffl) since we observed only one association (longer STL and decreased number of normal sperm cells) that is probably due to chance. The majority of the reports have a very limited sample size compared to our study and therefore could suffer from overinterpretation of the results. In addition, all the patients enrolled in this study are consecutive and therefore the chance of a bias in sampling the phenotypes are limited. This cannot be ascertained in all the other studies reported in the literature.

We tested, for the first time, the validity of the teloscore as a proxy of STL. We did not observe a strong association between the teloscore and STL, however two SNPs, *TERT*˗rs2736100, and *TERC˗*rs10936599, showed an effect on STL. Therefore, despite the different germinal cells levels of telomerase activity, we can suppose that the genetic variants *TERT*˗rs2736100 and *TERC˗*rs10936599 are associated with a specific telomerase regulation pathway which acts in the same way in both somatic and germinal cells. Despite the significance being weak, the effect of both SNPs is in line with what observed in studies conducted in LTL [40,42].

These are promising results, considering our sample size, and suggest that the 11 SNPs that are used in the teloscore could be considered as genetic proxies also in STL. However, it could also be possible that only *TERT*˗rs2736100 and *TERC˗*rs10936599 are involved in telomere length determination in both cell types, suggesting other genes and polymorphisms to determine telomere length in spermatozoa. Larger studies with a specific focus in finding genetic determinant of STL are needed to further our knowledge.

Considering the individual SNPs, we observed 5 statistically significant associations with sperm parameters. *PXK*˗rs6772228 is associated with the number of spermatozoa in the ejaculate is an eQTL for the *PXK* gene in the human testis. This SNP and the *PXK* gene are associated with rheumatoid arthritis and the systemic lupus erythematosus (SLE), that are related to the male fertility. Several studies have described the presence of anti-sperm antibodies in patients with SLE as the possible biological explanation of the impairment of spermatogenesis in these patients and the reduced sperm count [7,51,52,53,54]. *ACYP2*˗rs11125529 is associated with sperm motility expressed as total motility, progressive motility, and mot_ABCD score. This SNP is an eQTL of the *TSPYL6* gene and the A allele decreases the expression of the *TSPYL6* gene in the testis. The *TSPY* genes are a superfamily that includes *TSPYL1*, *TSPYL2*, *TSPYL3*, *TSPYL4*, and *TSPYL5*. There are no studies reporting a biological explanation of the relationship between the *TSPYL6* gene and male infertility, but two studies demonstrated that allelic variants of the *TSPYL1* gene might be associated with isolated gonadal dysgenesis or anomalies of the spermatogenesis that could cause abnormal production of spermatozoa [55,56]. It is possible to hypothesize that an altered spermatogenesis could also generate non-functional and less mobile spermatozoa. *NAF1*˗rs7675998 is associated with the majority of the morphological sperm parameters, including the normal morphology of the acrosome and flagellum. This SNP is an eQTL and a sQTL for the *NAF1* gene in the testis with the A allele associated with an increased expression of the gene. In a recent study, Iosub-Amir and colleagues reported that the NAF1 protein regulates apoptosis through the interaction with the iASPP peptide. It is possible that the increase of the NAF1 gene expression induces the apoptosis that in turn could favor the elimination of defective spermatozoa [57]. In our study, we observed that the A allele of rs7675998 is associated with an increased percentage of normal sperm suggesting that activation of the apoptotic process could increase the percentage of normal spermatozoa.

Finally, *ZNF208*-rs8105767 was associated with the percentage of spermatozoa with normal heads and with ABCD_mot score. The G allele (minor allele) of this SNP decreases the percentage of spermatozoa with normal heads, decreases the sperm motility and decreases the expression of *ZNF676* in the testis. The *ZNF676* gene was reported as a risk locus in nonobstructive azoospermia by means of a Transcriptome-wide association study (TWAS) in a Chinese population [58]. The authors also observed that *ZNF676* is co-expressed with the *ZNF678* gene, where lies a risk locus for azoospermia [59]. In their study Jiang and colleagues observed that genes co-expressed with *ZNF676* were enriched in pathways of spermatogenesis and male gamete generation, suggesting a central role in regulating these two pathways [58]. Our observation that *ZNF208*˗rs8105767 is associated with sperm parameters suggests that it could be instrumental in regulating the expression of *ZNF676* in human testicular tissue, although functional studies to better understand the role of *ZNF676* are warranted.

One of the strengths of this study is the number of patients enrolled; with 599 individuals, this is one of the largest studies in the field of TL in male infertility and certainly the largest among the studies carried out in Caucasian population. A potential limitation of this study is its generalizability because all the subjects are Caucasian and in addition all the functional data have been obtained by web tools, and in vitro studies were not performed.

## 4. Materials and Methods

### 4.1. Study Design and Populations

This association study was conducted in a total of 599 consecutive Caucasian male individuals undergoing semen evaluation at the Centre of Couple Sterility of the Siena University Hospital, between November 2013 and December 2016. For all subjects, information on age and smoking habits was collected (Table S2). The median age of the patients was 36 years (range: 18–59), and 192 (32%) were smokers. A comprehensive clinical history of patients was obtained, and most common causes of male infertility were excluded: genetic causes (chromosomal and gene abnormalities, e.g., CFTR mutations and Y microdeletions s), the congenital bilateral absence of vas deferens, varicocele, cryptorchidism, or endocrine disorders (out of range value for FSH, LH, and testosterone).

The study protocol was approved by the ethics commission of the Siena University Hospital (C.E.A.V.S.E., 19 November 2013) and all participants signed a written informed consent.

### 4.2. Characterization of Sperm Parameters

Seminal fluid was collected by masturbation after 2–4 days of sexual abstinence, left to liquefy at RT for 30 min, and then analyzed according to World Health Organization protocol [4]. The following sperm variables were evaluated: sperm concentration, sperm total number, progressive motility, no-progressive motility, total motility, sperm morphology, acrosome head, and flagellum morphology. In order to purify sperm from debris and any other somatic cells, a density gradient centrifugation by using a 80−40% Pure Sperm gradient (Nidacon, Mölndal, Sweden) was performed.

### 4.3. DNA Extraction

DNA was extracted from density gradient purified sperm samples and buccal cells collected by a brush for cell sampling, by using the Wizard^®^ Genomic DNA Purification Kit (Promega, Milan, Italy), according to the manufacturer protocol.

### 4.4. q-PCR Measurement of Sperm Telomere Length

The relative STL was measured in 239 DNA sperm samples because the remaining study subjects did not have sufficient sperm DNA. The measurement was based on the ratio in each sample between a nuclear reference gene as *Albumin* (*ALB*) according to Cawthon 2009 and Hosen 2015 [60,61] and the telomere repeat copy number (*Tel*), with two amplification reactions in the same volume. For each DNA sample, the qPCR was performed in triplicate, in an optical 96-well reaction plate, in a 20 µL reaction volume using 2 µL of 5X HOT FIREPol Probe qPCR Mix Plus with ROX (Solis Bio-Dyne, Tartu, Estonia), 1.5 µM of Syto 9 (Invitrogen, Carlsbad, CA, USA), 5 ng of genomic DNA, and 8 µL of water. The *ALB* primers were modified, adding a GC-clamp to the 5′ end in order to raise the melting temperature. The real-time PCR was carried out using a CFX96 Touch Real-Time PCR Detection System (Bio-Rad, Hercules, CA, USA) using two subsequent (#1 *Tel*; #2 *ALB*) PCR cycling conditions performed in the same plates, to acquire the respective cycle thresholds (Ct) values for copy numbers of *Tel* and *ALB* (control) gene. The primers for amplification of the *Albumin* gene are: Albugcr2CGGCGGCGGGCGGCGCGGGCTGGGCGGCCATGCTTTTCAGCTCTGCAAGTC and Albdgcr2: GCCCGGCCCGCCGCGCCCGTCCCGCCGAGCATTAAGCTCTTTGGCAACGTAGGTTTC. The predicted product size is 98 bp. The primers for amplification of the telomere repeat copy number are: TelgACACTAAGGTTTGGGTTTGGGTTTGGGTTTGGGTTAGTGT and Telc: TGTTAGGTATCCCTATCCCTATCCCTATCCCTATCCCTAACA. The predicted product size is 79 bp. A serial dilution (1:2) from 20 ng to 0.3 ng of genomic DNA pooled from 50 randomly chosen individuals were included to generate the standard curves for *Tel* and *ALB* genes. The standard curve was used to quantify the *Tel* repeats and *ALB* gene, based on the respective Ct values. For each data point, the obtained triplicate values were averaged. Individual values that deviated from the average of the triplicates by more than 5% of the standard deviation were discarded. STL was expressed as the ratio between *Tel*/*ALB*, using the Pfaffl method [49], which is best suited to the type of data obtained from a qPCR with efficiencies not perfectly identical between the amplification reactions of *Tel* and *ALB*, using as a calibrator the Ct of the standard curve to the equivalent of 5 ng of DNA.

### 4.5. SNP Selection and Genotyping

Eleven SNPs associated with telomere length (*ZNF676*-rs412658, *TERT*-rs2736100, *CTC1*-rs3027234, *DHX35*-rs6028466, *PXK*-rs6772228, *NAF1*-rs7675998, *ZNF208*-rs8105767, *OBFC1*-rs9420907, *ACYP2*-rs11125529, *TERC*-rs10936599, and *ZBTB46*-rs755017) were genotyped [40,41,42,62]. Characteristics of the SNPs are summarized in Table S4.

### 4.6. Genotyping and Quality Control

Genotyping was performed, in 384 well plates, using the allele specific TaqMan (ABI, Applied Biosystems, Foster City, CA, USA) technology according to the manufacturer’s recommendations. Each 384 well plate was prepared to contain a minimum of 12 (14%) negative controls and around 8% of the samples were duplicated for quality controls. The amplification reaction was performed using a Verity thermal cycler (Applied Biosystems, Foster City, CA, USA) and the following genotype detection was performed using a QuantStudio 5 Real-Time PCR Systems (Applied Biosystems, Foster City, CA, USA). The average SNPs call rate was 98% (93−100%) and all polymorphisms were in Hardy-Weinberg equilibrium. After quality control, 14 subjects (2.4%) were discarded because they had a call rate lower than 80% and 494 subjects had 100% SNP call rate. The concordance rate with duplicated samples was higher than 99%.

### 4.7. Teloscore Computation

We built an unweighted score and a weighted score, as previously reported [47]. Briefly, the unweighted score was computed for each subject, from the result of the sum of the genotypes converted in numerical value based on the number of alleles associated with longer telomere, 0 for the homozygous without effect alleles, 1 for the heterozygous, and 2 for the homozygous with two effect alleles. Therefore, with 11 SNPs, the unweighted score could assume values between 0 (shortest telomeres) and 22 (longest telomeres). In addition to the unweighted score, a weighted score was also computed considering the estimates of the effect of each allele. The effect for each allele was taken by the literature to avoid overfitting. Additional information on the computation of the score have been given elsewhere [47]. After genotyping, 585 subjects had a call-rate >80% (~9 SNPs of 11) and only a subset of the study subjects had 100% SNP call rate (494 subjects). Therefore, to be able to compute comparable score values for all study subjects, we also considered a scaled score for each of 585 subjects with a call-rate >80% (dividing the obtained score for the number of genotyped SNPs and multiplying for 11) to obtain scaled unweighted and weighted scores.

### 4.8. Statistical Analysis

We used linear regression models to explore the associations between the telomere length measures (scores; single SNPs; and STL) and the sperm parameters. The analyses were adjusted for age (continuous variable expressed in years) and smoking status (yes/no). STL, quantified through the Pfaffl’s method, was log-transformed to attain approximate normality. In the regression linear models where log STL was the outcome variable we included reaction plate as random effect. To remove the effect of plates in models where STL was the independent variable, we used the residuals of the regression of log STL on the plates. When testing the association between the SNPs and sperm parameters, we used the additive and co-dominant models of inheritance with the major allele used as a reference. We analyzed the TL genetic score and the STL value as continuous variables and categorized in quintiles, using the first quintile as a reference group. To reduce the number of parameters describing the sperm quality, we computed the “ABCD score” using four sperm parameters: sperm concentration, progressive motility, total motility, and sperm morphology.

First, we created a pseudo continuous variable for each of these parameters based on their density distribution, assigning l. Then, the total ABCD score was obtained as the total of the 4 scores. In a similar way we computed a motility ABCD (ABCD_mot) score, using the progressive motility and the total motility. All statistical tests were two-sided, at a significance level of 0.05/33 (0.0015) considering 11 SNPs and three models of inheritance. All statistical analyses were performed using R (version 3.6.2).

### 4.9. Bioinformatic Analysis

To identify the possible effect of the SNPs on the expression of nearby genes, we used the data available in the GTEx website (http://www.gtexportal.org/home/) (Access Date: 1 March 2020) and RegulomeDB (http://regulomedb.org/) (Access Date: 1 March 2020) to explore potential regulatory functions for the SNPs that showed a significant association with the sperm parameters.

## 5. Conclusions

Our result suggests a moderate involvement of telomere length in male infertility and although several studies indicate telomere length as a risk factor for male infertility, no one has a biological explanation for this link. Even though we do not find a clear connection between telomere and male infertility in our analyses, four SNPs are weakly associated with sperm variables, suggesting these SNPs to be pleiotropic and to be involved in other regulatory mechanisms independent from telomere homeostasis, but involved in the spermatogenic process. We observed several associations between the SNPs and male infertility with plausible biological explanation, however we have conducted many analyses and the *p* values that we obtain are borderline and could reflect statistical fluctuation. In conclusion, the results of our study suggest the lack of direct involvement of telomere length with male infertility but the possible involvement of the selected SNPs through other mechanisms.

## Figures and Tables

**Table 1:** Age and seminal characteristics of enrolled patients.

	Total	ABCD Groups			
		A (<5th)	B (5–50th)	C (50–95th)	D (>95th)
Age (years)	34.8 (±7.5) (*n* = 599)				
Sperm concentration (10^6^/mL)	55.4 (±49.2)	6.7 (±4.5) (*n* = 155)	38.8 (±16.5) (*n* = 275)	114.5 (±32.1) (*n* = 162)	256 (±30.1) (*n* = 7)
Progressive motility (%)	45.9 (±18.1)	18.8 (±10.1) (*n* = 121)	44.0 (±8.0) (*n* = 246))	61.1 (±8.3) (*n* = 214)	74.4 (±1.9) (*n* = 18)
Total motility (%)	48.3 (±16.8)	26.3 (±11.4) (*n* = 152)	50.0 (±12.3) (*n* = 279)	64.9 (±7.5) (*n* = 159)	78.2 (±7.9) (*n* = 8)
Sperm morphology (%)	7.4 (±4.3)	2.9 (±1.7) (*n* = 105)	7.9 (±3.7) (*n* = 435)	16.4 (±2.7) (*n* = 54)	19.2 (±1.5) (*n* = 5)

Data are expressed as value (±SD) (number of patients).

**Table 2:** Associations between age and sperm parameters.

	Univariable Model		Multivariable Model *	
Sperm Parameters	Coefficient (95% CI)	*p*	Coefficient (95% CI)	*p*
Log STL	0.006 (−0.004 to 0.015)	0.25	0.005 (−0.005 to 0.016)	0.31
Concentration	0.23 (−0.28 to 0.74)	0.38	0.13 (−0.39 to 0.65)	0.63
Total number	0.12 (−1.54 to 1.78)	0.89	−0.09 (−1.83 to 1.64)	0.91
Progressive motility	−0.31 (−0.50 to −0.13)	1.00 × 10^−3^	−0.31 (−0.50 to −0.11)	2.00 × 10^−3^
Non-progressive motility	0.06 (0.00 to 0.12)	0.07	0.04 (−0.01 to 0.09)	0.09
Total motility	−0.28 (−0.46 to −0.11)	2.00 × 10^−3^	−0.31 (−0.49 to −0.13)	1.00 × 10^−3^
Normal acrosome	−0.08 (−0.14 to −0.02)	0.01	−0.07 (−0.14 to −0.01)	0.03
Normal head	−0.13 (−0.23 to −0.03)	0.01	−0.12 (−0.22 to −0.01)	0.03
Normal flagella	−0.15 (−0.26 to −0.05)	4.00 × 10^−3^	−0.15 (−0.26 to −0.04)	8.00 × 10^−3^
ABCD score	−0.03 (−0.05 to 0.00)	0.05	−0.03 (−0.06 to 0.00)	0.03
ABCD_mot score	−0.03 (−0.04 to −0.01)	2.00 × 10^−3^	−0.03 (−0.05 to −0.01)	1.00 × 10^−3^
ABCD_mot score	−0.03 (−0.04 to −0.01)	2.00 × 10^−3^	−0.03 (−0.05 to −0.01)	1.00 × 10^−3^

Linear regressions of age on log STL and the sperm parameters. In the model for log STL, reaction plate is included as random effect. * Analyses adjusted for smoking status.

**Table 3:** Association between sperm telomere length (STL) and sperm parameters.

	Log STL Residuals (Linear)	2nd vs. 1st Quintile	3rd vs. 1st Quintile	4th vs. 1st Quintile)	5th vs. 1st Quintile
Sperm Parameters	Coeff. (95% CI)	Coeff. (95% CI)	Coeff. (95% CI)	Coeff. (95% CI)	Coeff. (95% CI)
Concentration	−1.44 (−12.38–9.50)	0.34 (−19.75–20.42)	5.91 (−13.80–25.62)	−2.55 (−22.62–17.51)	6.95 (−12.55–26.44)
Total number	−3.09 (−39.91–33.7)	−7.37 (−74.93–60.19)	23.72 (−42.59–90.02)	0.66 (−66.85–68.17)	25.16 (−40.43–90.74)
Progressive motility	−0.42 (−4.5–3.65)	−1.19 (−8.67–6.30)	−2.47 (−9.82–4.87)	−2.60 (−10.08–4.88)	0.94 (−6.33–8.2)
Non-progressive motility	0.19 (−0.66–1.04)	−0.98 (−2.54–0.58)	−0.63 (−2.17–0.90)	−0.83 (−2.39–0.73)	−0.27 (−1.78–1.25)
Total motility	−1.80 (−5.64–2.03)	0.00 (−7.05–7.06)	−2.13 (−9.06–4.79)	−2.48 (−9.53–4.57)	0.26 (−6.59–7.11)
Normal morphology	−0.30 (−1.48–0.88)	−1.71 (−3.86–0.43)	−1.61 (−3.72–0.50)	−2.25 * (−4.40–−0.11)	−0.81 (−2.89–1.28)
Normal acrosome	0.08 (−1.33–1.48)	−0.19 (−2.78–2.41)	0.21 (−2.32–2.73)	−1.64 (−4.21–0.92)	0.23 (−2.28–2.74)
Normal head	−0.92 (−3.18–1.34)	0.77 (−3.28–4.81)	1.14 (−2.79–5.08)	−1.88 (−5.88–2.12)	0.69 (−3.24–4.61)
Normal flagellum	−1.80 (−4.23–0.63)	−0.97 (−5.33–3.39)	−1.60 (−5.85–2.64)	−3.74 (−8.06–0.58)	−1.49 (−5.73–2.75)
ABCD	−0.24 (−0.74–0.26)	0.14 (−0.77–1.06)	−0.18 (−1.07–0.72)	−0.19 (−1.11–0.72)	0.09 (−0.80–0.97)
ABCD_mot	−0.15 (−0.50–0.20)	0.10 (−0.55–0.74)	−0.16 (−0.79–0.47)	−0.25 (−0.89–0.39)	−0.08 (−0.71–0.54)

Linear regressions of log STL on the sperm parameters. Analyses are adjusted for age and smoking status. * *p* = 0.04.

**Table 4:** Associations between the teloscore and the single nucleotide polymorphisms (SNPs) with log-transformed sperm telomere length (STL).

	Coefficient (95% CI)	*p*
U-GS (linear)	0.01 (−0.03–0.04)	0.60
2nd vs. 1st quintile	−0.05 (−0.27–0.18)	0.69
3rd vs. 1st quintile	0.06 (−0.17–0.30)	0.60
4th vs. 1st quintile	0.08 (−0.14–0.30)	0.50
5th vs. 1st quintile	0.07 (−0.19–0.33)	0.60
W-GS (linear)	1 × 10^−4^ (−4 × 10^−4^–6 × 10^−4^)	0.76
2nd vs. 1st quintile	−0.06 (−0.30–0.18)	0.64
3rd vs. 1st quintile	−0.09 (−0.33–0.15)	0.47
4th vs. 1st quintile	−0.08 (−0.33–0.16)	0.50
5th vs. 1st quintile	0.03 (−0.21–0.27)	0.79
rs11125529	0.18 (−0.02–0.37)	0.07
C/A vs. C/C	0.19 (−0.04–0.43)	0.10
A/A vs. C/C	0.29 (−0.38–0.96)	0.39
rs3027234	0.01 (−0.13–0.14)	0.91
C/T vs. C/C	−0.06 (−0.24–0.12)	0.50
T/T vs. C/C	0.14 (−0.21–0.49)	0.42
rs6028466	−0.13 (−0.34–0.09)	0.25
G/A vs. G/G	−0.15 (−0.38–0.09)	0.22
A/A vs. G/G	−0.02 (−1.17–1.13)	0.97
rs7675998	−0.05 (−0.17–0.08)	0.47
G/A vs. G/G	−0.04 (−0.20–0.12)	0.61
A/A vs. G/G	−0.10 (−0.43–0.22)	0.54
rs9420907	−0.09 (−0.23–0.06)	0.25
A/C vs. C/C	−0.16 (−0.33–0.01)	0.06
A/A vs. C/C	0.12 (−0.33–0.56)	0.60
rs6772228	0.20 (−0.19–0.58)	0.32
T/A vs. T/T	0.20 (−0.19–0.58)	0.32
rs10936599	−0.10 (−0.23–0.03)	0.14
C/T vs. C/C	−0.17 (−0.33–−0.01)	0.04
T/T vs. C/C	0.01 (−0.38–0.39)	0.97
rs2736100	0.11 (0.003–0.22)	0.05
C/A vs. C/C	0.12 (−0.06–0.30)	0.19
A/A vs. C/C	0.22 (0.001–0.44)	0.05
rs755017	0.10 (−0.10–0.30)	0.31
A/G vs. A/A	0.10 (−0.10–0.30)	0.31
rs8105767	0.08 (−0.04–0.20)	0.17
A/G vs. A/A	0.08 (−0.08–0.25)	0.32
G/G vs. A/A	0.16 (−0.12–0.44)	0.25
rs412658	0.06 (−0.05–0.17)	0.32
C/T vs. C/C	−0.03 (−0.20–0.13)	0.69
T/T vs. C/C	0.17 (−0.06–0.41)	0.15

For the SNPs the additive (first line for each SNP) and codominant (second and third lines for each SNPs) inheritance models were used, with the more common allele as reference. U-GS: unweighted genetic score; W-GS: weighted genetic score.

**Table 5:** Statistically significant associations of log-transformed STL, teloscores, and SNPs with sperm parameters.

	Coefficient (95% CI)	*p*	Coefficient (95% CI)	*p*	Coefficient (95% CI)	*p*
Unweighted genetic score	normal acrosome	-	*-*	*-*	*-*	*-*
2nd vs. 1st quintile	−1.26 (−2.88–0.36)	0.13	-	*-*	-	*-*
3rd vs. 1st quintile	−0.64 (−2.21–0.94)	0.43	-	*-*	-	*-*
4th vs. 1st quintile	−0.12 (−1.64–1.41)	0.88	-	*-*	-	*-*
5th vs. 1st quintile	−1.90 (−3.74–−0.05)	0.04	-	*-*	-	*-*
Weighted genetic score	normal head	-	*-*	*-*	*-*	*-*
2nd vs. 1st quintile	−2.79 (−5.40–−0.17)	0.04	-	*-*	-	*-*
3rd vs. 1st quintile	−1.22 (−3.90–1.47)	0.37	-	*-*	-	*-*
4th vs. 1st quintile	−1.28 (−3.88–1.31)	0.33	-	*-*	-	*-*
5th vs. 1st quintile	−1.78 (−4.44–0.88)	0.19	-	*-*	-	*-*
STL (quintiles)	normal morphology	-	*-*	*-*	*-*	*-*
2nd vs. 1st quintile	−1.71 (−3.86–0.43)	0.12	-	*-*	-	*-*
3rd vs. 1st quintile	−1.61 (−3.72–0.50)	0.14	-	*-*	-	*-*
4th vs. 1st quintile	−2.25 (−4.40–−0.11)	0.04	-	*-*	-	*-*
5th vs. 1st quintile	−0.81 (−2.89–1.28)	0.45	-	*-*	-	*-*
rs11125529	total motility	-	ABCD	*-*	ABCD_mot	-
additive model	4.16 (0.52–7.80)	0.03	0.56 (0.02–1.10)	0.04	0.38 (0.04–0.72)	0.03
C/A vs. C/C	4.08 (0.08–8.08)	0.05	0.59 (0.00–1.18)	0.05	0.37 (0.00–0.75)	0.05
A/A vs. C/C	9.06 (−7.43–25.55)	0.28	0.86 (−1.60–3.32)	0.49	0.82 (−0.75–2.39)	0.31
rs7675998	normal acrosome	-	normal flagellum	-	-	*-*
additive model	0.86 (−0.02–1.74)	0.06	1.04 (−0.40–2.48)	0.16	-	*-*
G/A vs. G/G	0.46 (−0.66–1.58)	0.42	0.12 (−1.71–1.95)	0.90	-	*-*
A/A vs. G/G	2.54 (0.26–4.81)	0.03	4.00 (0.29–7.72)	0.04	-	*-*
rs6772228	sperm concentration	-	total number	-	-	*-*
additive model	26.31 (4.52–48.09)	0.02	76.24 (3.25–149.23)	0.04	-	*-*
T/A vs. T/T	26.31 (4.52–48.09)	0.02	76.24 (3.25–149.23)	0.04	-	*-*
rs8105767	normal head	-	ABCD_mot	-	-	*-*
additive model	−0.98 (−2.29–0.34)	0.14	−0.08 (−0.30–0.14)	0.49	-	*-*
A/G vs. A/A	−2.39 (−4.16–−0.61)	0.01	−0.37 (−0.66–−0.07)	0.01	-	*-*
G/G vs. A/A	0.00 (−3.10–3.11)	1.00	0.27 (−0.25–0.79)	0.31	-	*-*

Analyses are adjusted for age and smoking behavior. For SNPs the additive and codominant inheritance models are used, with the more common allele as reference. U-GS: unweighted genetic score; W-GS: weighted genetic score. For STL, plate was used as additional adjustment.

**Table 6:** ABCD scores association analyses results.

	ABCD		ABCD_mot	
	Coefficient (95% CI)	*p*	Coefficient (95% CI)	*p*
rs11125529	0.56 (0.02–1.10)	0.04	0.38 (0.04–0.72)	0.03
C/A vs. C/C	0.59 (0.00–1.18)	0.05	0.37 (0.00–0.75)	0.05
A/A vs. C/C	0.86 (−1.60–3.32)	0.49	0.82 (−0.75–2.39)	0.31
rs3027234	0.03 (−0.34–0.41)	0.86	−0.01 (−0.25–0.23)	0.94
C/T vs. C/C	0.25 (−0.22–0.73)	0.30	0.17 (−0.13–0.47)	0.28
T/T vs. C/C	−0.43 (−1.42–0.57)	0.40	−0.41 (−1.04–0.22)	0.20
rs6028466	−0.2 (−0.86–0.46)	0.56	−0.03 (−0.45–0.38)	0.88
G/A vs. G/G	−0.2 (−0.91–0.50)	0.58	−0.03 (−0.48–0.41)	0.88
A/A vs. G/G	−0.31 (−3.81–3.20)	0.86	−0.04 (−2.27–2.19)	0.97
rs7675998	0.04 (−0.32–0.39)	0.83	0.01 (−0.21–0.23)	0.93
G/A vs. G/G	0.07 (−0.39–0.53)	0.77	0.04 (−0.25–0.34)	0.76
A/A vs. G/G	0.02 (−0.87–0.91)	0.96	−0.04 (−0.61–0.52)	0.88
rs9420907	−0.25 (−0.65–0.15)	0.23	−0.18 (−0.43–0.08)	0.18
A/C vs. C/C	−0.16 (−0.66–0.33)	0.52	−0.07 (−0.39–0.25)	0.66
A/A vs. C/C	−0.75 (−1.92–0.43)	0.21	−0.66 (−1.40–0.09)	0.09
rs6772228	0.83 (−0.33–1.98)	0.16	0.62 (−0.12–1.35)	0.10
T/A vs. T/T	0.83 (−0.33–1.98)	0.16	0.62 (−0.12–1.35)	0.10
rs10936599	−0.10 (−0.49–0.28)	0.60	−0.06 (−0.31–0.18)	0.60
C/T vs. C/C	−0.27 (−0.74–0.20)	0.26	−0.18 (−0.47–0.12)	0.24
T/T vs. C/C	0.29 (−0.81–1.39)	0.60	0.21 (−0.48–0.91)	0.55
rs2736100	−0.06 (−0.38–0.27)	0.72	−0.08 (−0.29–0.12)	0.44
C/A vs. C/C	−0.08 (−0.60–0.44)	0.77	−0.14 (−0.47–0.19)	0.41
A/A vs. C/C	−0.11 (−0.77–0.54)	0.74	−0.15 (−0.56–0.27)	0.49
rs755017	0.43 (−0.15–1.01)	0.15	0.26 (−0.11–0.62)	0.17
A/G vs. A/A	0.36 (−0.23–0.95)	0.23	0.19 (−0.19–0.57)	0.32
G/G vs. A/A	3.34 (−1.61–8.28)	0.19	3.04 (−0.10–6.18)	0.06
rs8105767	0.00 (−0.35–0.35)	1.00	−0.08 (−0.30–0.14)	0.49
A/G vs. A/A	−0.38 (−0.84–0.08)	0.11	−0.37 (−0.66–−0.07)	0.01
G/G vs. A/A	0.57 (−0.26–1.40)	0.18	0.27 (−0.25–0.79)	0.31
rs412658	−0.10 (−0.43–0.22)	0.54	−0.09 (−0.3–0.11)	0.37
C/T vs. C/C	−0.37 (−0.85–0.10)	0.12	−0.30 (−0.60–0.00)	0.05
T/T vs. C/C	0.00 (−0.70–0.70)	1.00	−0.04 (−0.48–0.40)	0.86

Analyses are adjusted for age and smoking status, using an additive and codominant inheritance model, and using the more common allele as reference. ABCD_mot: ABCD score computed using only the motility variables.

**Table 7:** Annotation reported in bioinformatics web-tools for the seven suggestive SNPs.

Sperm Parameter	SNP	M/m	Pos.38	Gene	Reg.DB	GTEx	R/A	Gene Symbol	*p*-Value	NES	Tissue
nH;ABCD_mot	rs8105767	A/G	19:22032639	*LOC112268248*	1f	s/eQTL	A/G	*ZNF676*	1.20 × 10^−11^	−0.36	Testis
						eQTL		*RP11-157B13.7*	1.70 × 10^−8^	−0.2	Testis
						eQTL		*ZNF729*	1.00 × 10^−4^	−0.21	Testis
nA;nF	rs7675998	G/A	4:163086668	None	6	eQTL	A/G	*RP11-563E2.2*	-	−0.25	no-testis
						eQTL		*NAF1*	-	[+/-]*	no-testis
						sQTL		*NAF1*	1.50 × 10^−6^	0.31	no-testis
TM; ABCD; ABCD_mot	rs11125529	C/A	2:54248729	*ACYP2*: Intron Variant	5	eQTL	C/A	*TSPYL6*	1.80 × 10^−69^	−0.95	Testis
SC, STn	rs6772228	T/A	3:58390292	*PXK*: Intron Variant	7	sQTL	T/A	*PXK*	1.70 × 10^−16^	1.40	Testis
						eQTL		*FAM107A*			no-testis
						eQTL		*PDHB*			no-testis
						eQTL		*RP11-359I18.5*			no-testis
						eQTL		*RPP14*			no-testis
STL	rs10936599	C/T	3:169774313	*MYNN*: Missense Variant	3a	eQTL	C/T	*MYNN*	2.10 × 10^−11^	0.35	Testis
								*LRRIQ4*	1.90 × 10^−6^	0.19	Testis
STL	rs2736100	C/A	5:1286401	*TERT*: Intron Variant	5	eQTL	C/A	*TERT*	4.80 × 10^−5^	0.23	no-testis

Sparameter: sperm parameters associated with a SNP in our study; M/m: major and minor allele; Pos.38: chromosomic position in (human) genome assembly GRCh38 (hg38); Reg.DB: score reported in RegulomeDB dataset; R/A: reference and alternative allele; NES: normalized effect size. (*) The rs7675998 was an eQTL of *NAF1* gene with positive NEF in Cells—Cultured fibroblasts, and negative NEF in other four tissues. Data obtained from the GTEx Portal on 05/01/20. ABCD_mot: score composed using motility parameters; TM: total motility; PM: progressive motility; SC: sperm concentration; STn: sperm total number; STL: sperm telomere length; nH: normal head; nA: normal Acrosome; nF: normal flagellum.

## Data Availability

Data are available at request from the authors.

## Supplementary Materials

The following are available online at https://www.mdpi.com/article/10.3390/ijms22083959/s1, Table S1: Association between teloscore and sperm parameters; Table S2: Association between individual SNPs and sperm parameters; Table S3: Study population description; Table S4: SNPs reported to be associated with telomere length by previously published GWAS.
