# Supplementary material for: Telomere Length and Male Fertility
Source: Int J Mol Sci. 2021 Apr 12;22(8):3959. doi: 10.3390/ijms22083959 (PMC8069448; doi:10.3390/ijms22083959)
Supplement: Supplementary file 1 [file ijms-22-03959-s001.zip › Supplementary table/Supplementary Table AI.docx]

**Supplementary table AI:** Association between teloscore and sperm parameters

|  | **U-GS (linear)** | | **2^nd^ vs 1^st^ quintile** | | **3^rd^ vs 1^st^ quintile** | | **4^th^ vs 1^st^ quintile** | | **5^th^ vs 1^st^ quintile** | |
| --- | --- | --- | --- | --- | --- | --- | --- | --- | --- | --- |
| **Outcome** | **Coeff. (95% CI)** | **p** | **Coeff. (95% CI)** | **p** | **Coeff. (95% CI)** | **p** | **Coeff. (95% CI)** | **p** | **Coeff. (95% CI)** | **p** |
| Sperm concentration | 0.352 (-1.622˗2.326) | *0.73* | -11.76 (-24.41˗0.89) | *0.07* | 0.90 (-11.22˗13.02) | *0.88* | 0.97 (-10.87˗12.80) | *0.87* | -3.66 (-18.15˗10.83) | *0.62* |
| Total number | -1.013 (-7.617˗5.591) | *0.76* | -10.16 (-52.62˗32.3) | *0.64* | 11.36 (-29.34˗52.05) | *0.58* | -2.53 (-42.26˗37.20) | *0.90* | -12.86 (-61.52˗35.80) | *0.60* |
| progressive motility | 0.134 (-0.591˗0.860) | *0.72* | 1.70 (-2.97˗6.36) | *0.48* | 2.06 (-2.41˗6.53) | *0.37* | 1.74 (-2.62˗6.10) | *0.43* | -0.53 (-5.88˗4.81) | *0.85* |
| non-progressive motility | -0.056 (-0.232˗0.119) | *0.53* | 0.03 (-1.10˗1.17) | *0.96* | 0.37 (-0.71˗1.45) | *0.51* | -0.01 (-1.07˗1.04) | *0.98* | -0.54 (-1.83˗0.76) | *0.42* |
| total motility | 0.101 (-0.594˗0.796) | *0.78* | 0.88 (-3.59˗5.35) | *0.70* | 2.68 (-1.60˗6.96) | *0.22* | 0.79 (-3.39˗4.97) | *0.71* | 0.52 (-4.60˗5.64) | *0.84* |
| normal morphology | 0.008 (-0.171˗0.188) | *0.93* | -0.54 (-1.68˗0.61) | *0.36* | 0.39 (-0.71˗1.49) | *0.49* | 0.51 (-0.57˗1.59) | *0.35* | -0.87 (-2.18˗0.45) | *0.20* |
| normal acrosome | -0.091 (-0.343˗0.162) | *0.48* | -1.26 (-2.88˗0.36) | *0.13* | -0.64 (-2.21˗0.94) | *0.43* | -0.12 (-1.64˗1.41) | *0.88* | -1.90 (-3.74˗-0.05) | ***0.04*** |
| normal head | -0.154 (-0.548˗0.239) | *0.44* | -1.81 (-4.33˗0.72) | *0.16* | -0.86 (-3.32˗1.59) | *0.49* | -0.58 (-2.98˗1.81) | *0.63* | -1.70 (-4.57˗1.18) | *0.25* |
| normal flagellum | -0.078 (-0.492˗0.335) | *0.71* | -1.75 (-4.40˗0.90) | *0.20* | -0.61 (-3.19˗1.97) | *0.64* | -0.77 (-3.29˗1.74) | *0.55* | -1.35 (-4.37˗1.67) | *0.38* |
| ABCD | 0.001 (-0.103˗0.105) | *0.98* | -0.21 (-0.87˗0.45) | *0.53* | -0.21 (-0.85˗0.42) | *0.51* | -0.11 (-0.73˗0.52) | *0.74* | -0.01 (-0.77˗0.76) | *0.98* |
| ABCD_mot | -0.001 (-0.067˗0.064) | *0.97* | -0.13 (-0.55˗0.30) | *0.56* | -0.13 (-0.53˗0.27) | *0.52* | -0.04 (-0.43˗0.36) | *0.86* | -0.03 (-0.51˗0.46) | *0.91* |
|  |  |  |  |  |  |  |  |  |  |  |
| **Outcome** | **W-GS (linear)** |  | **2^nd^ vs 1^st^ quintile** | | **3^rd^ vs 1^st^ quintile** | | **4^th^ vs 1^st^ quintile** | | **5^th^ vs 1^st^ quintile** | |
| Sperm concentration | 0.008 (-0.021˗0.038) | *0.59* | -8.12 (-21.16˗4.92) | *0.22* | -6.05 (-19.30˗7.20) | *0.37* | 6.72 (-6.14˗19.58) | *0.31* | -6.27 (-19.46˗6.93) | *0.35* |
| Total number | -0.014 (-0.113˗0.084) | *0.78* | 0.96 (-42.82˗44.73) | *0.97* | -13.52 (-58.01˗30.96) | *0.55* | 15.12 (-28.05˗58.29) | *0.49* | -22.87 (-67.17˗21.42) | *0.31* |
| progressive motility | 0.003 (-0.008˗0.014) | *0.55* | -1.25 (-6.06˗3.57) | *0.61* | 0.93 (-3.96˗5.83) | *0.71* | 1.74 (-3.01˗6.49) | *0.47* | 0.22 (-4.66˗5.09) | *0.93* |
| non-progressive motility | -0.001 (-0.004˗0.001) | *0.36* | 0.01 (-1.16˗1.18) | *0.99* | 0.43 (-0.76˗1.62) | *0.48* | -0.17 (-1.32˗0.99) | *0.78* | -0.18 (-1.37˗1.00) | *0.76* |
| total motility | 0.002 (-0.008˗0.012) | *0.72* | -1.03 (-5.64˗3.58) | *0.66* | 0.68 (-4.01˗5.37) | *0.78* | 1.85 (-2.7˗6.40) | *0.43* | -0.07 (-4.74˗4.60) | *0.98* |
| normal morphology | 0.000 (-0.002˗0.003) | *0.72* | -0.88 (-2.07˗0.30) | *0.15* | -0.37 (-1.58˗0.83) | *0.55* | 0.62 (-0.56˗1.79) | *0.30* | -0.40 (-1.6˗0.81) | *0.52* |
| normal acrosome | 0.000 (-0.004˗0.003) | *0.80* | -0.75 (-2.44˗0.93) | *0.38* | -0.79 (-2.52˗0.94) | *0.37* | 0.00 (-1.67˗1.66) | *1.00* | -1.11 (-2.81˗0.60) | *0.20* |
| normal head | -0.001 (-0.007˗0.005) | *0.70* | -2.79 (-5.40˗-0.17) | ***0.04*** | -1.22 (-3.90˗1.47) | *0.37* | -1.28 (-3.88˗1.31) | *0.33* | -1.78 (-4.44˗0.88) | *0.19* |
| normal flagellum | -0.001 (-0.007˗0.005) | *0.79* | -2.81 (-5.55˗-0.06) | *0.05* | -1.41 (-4.23˗1.40) | *0.33* | -1.02 (-3.74˗1.71) | *0.46* | -1.54 (-4.33˗1.25) | *0.28* |
| ABCD | 0.000 (-0.002˗0.002) | *0.99* | 0.01 (-0.68˗0.70) | *0.98* | -0.38 (-1.07˗0.31) | *0.28* | 0.26 (-0.42˗0.94) | *0.45* | -0.13 (-0.83˗0.56) | *0.71* |
| ABCD_mot | 0.000 (-0.001˗0.001) | *0.95* | 0.00 (-0.44˗0.43) | *0.99* | -0.28 (-0.71˗0.16) | *0.22* | 0.17 (-0.26˗0.60) | *0.44* | -0.05 (-0.49˗0.39) | *0.82* |

Linear regressions of the sperm parameters on weighted and unweighted teloscores. Analyses are adjusted for age and smoking status. **U-GS**: unweighted genetic score; **W-GS**: weighted genetic score.
