# Supplementary material for: Telomere Length and Male Fertility
Source: Int J Mol Sci. 2021 Apr 12;22(8):3959. doi: 10.3390/ijms22083959 (PMC8069448; doi:10.3390/ijms22083959)
Supplement: Supplementary file 1 [file ijms-22-03959-s001.zip › Supplementary table/Supplementary Table AII.docx]

**Supplementary table AII:** Association between individual SNPs and sperm parameters

|  | **Sperm concentration** | | **Total number** | | **Progressive motility** | | **Non-progressive motility** | | **Total motility** | | **Normal morphology** | | **Normal acrosome** | | **Normal head** | | **Normal flagellum** | |
| --- | --- | --- | --- | --- | --- | --- | --- | --- | --- | --- | --- | --- | --- | --- | --- | --- | --- | --- |
|  | Estimate (95% CI) | P | Estimate (95% CI) | P | Estimate (95% CI) | P | Estimate (95% CI) | P | Estimate (95% CI) | P | Estimate (95% CI) | P | Estimate (95% CI) | P | Estimate (95% CI) | P | Estimate (95% CI) | P |
| **rs11125529** | 3.62 (-6.77˗14.00) | 0.50 | 10.91 (-23.86˗45.69) | 0.54 | 3.80 (-0.01˗7.61) | 0.05 | 0.27 (-0.66˗1.19) | 0.57 | 4.16 (0.52˗7.80) | **0.03** | 0.51 (-0.43˗1.45) | 0.29 | 0.97 (-0.37˗2.30) | 0.16 | 0.69 (-1.39˗2.77) | 0.52 | 1.54 (-0.64˗3.72) | 0.17 |
| C/A vs C/C | 6.31 (-5.07˗17.69) | 0.28 | 12.01 (-26.15˗50.16) | 0.88 | 3.63 (-0.55˗7.81) | 0.09 | 0.38 (-0.64˗1.39) | 0.47 | 4.08 (0.08˗8.08) | 0.05 | 0.66 (-0.38˗1.69) | 0.21 | 1.32 (-0.14˗2.79) | 0.08 | 0.38 (-1.91˗2.67) | 0.75 | 1.16 (-1.24˗3.56) | 0.34 |
| A/A vs C/C | -17.10 (-64.05˗29.85) | 0.48 | 11.93 (-145.47˗169.34) | 0.26 | 9.12 (-8.12˗26.36) | 0.30 | -0.45 (-4.64˗3.74) | 0.83 | 9.06 (-7.43˗25.55) | 0.28 | -0.30 (-4.56˗3.96) | 0.89 | -1.16 (-7.10˗4.78) | 0.70 | 4.08 (-5.18˗13.34) | 0.39 | 6.36 (-3.34˗16.06) | 0.20 |
| **rs3027234** | 1.04 (-6.16˗8.24) | 0.78 | 13.58 (-10.23˗37.38) | 0.26 | -1.12 (-3.74˗1.49) | 0.40 | -0.01 (-0.64˗0.61) | 0.97 | -1.21 (-3.72˗1.29) | 0.34 | 0.12 (-0.53˗0.77) | 0.72 | -0.55 (-1.45˗0.34) | 0.23 | 0.11 (-1.32˗1.53) | 0.88 | 0.15 (-1.32˗1.62) | 0.84 |
| C/T vs C/C | 0.87 (-8.28˗10.02) | 0.85 | 18.58 (-11.67˗48.84) | 0.23 | -0.04 (-3.36˗3.28) | 0.98 | 0.02 (-0.77˗0.82) | 0.95 | 0.19 (-2.98˗3.37) | 0.91 | 0.53 (-0.30˗1.36) | 0.21 | -0.47 (-1.61˗0.68) | 0.43 | 0.37 (-1.46˗2.19) | 0.69 | 0.80 (-1.08˗2.68) | 0.40 |
| T/T vs C/C | 2.45 (-16.86˗21.76) | 0.80 | 15.75 (-48.09˗79.59) | 0.63 | -4.71 (-11.72˗2.30) | 0.19 | -0.10 (-1.77˗1.58) | 0.91 | -5.63 (-12.33˗1.07) | 0.10 | -0.69 (-2.43˗1.06) | 0.44 | -1.29 (-3.67˗1.08) | 0.29 | -0.35 (-4.13˗3.42) | 0.85 | -1.13 (-5.02˗2.76) | 0.57 |
| **rs6028466** | -2.77 (-15.43˗9.89) | 0.67 | -12.41 (-54.73˗29.92) | 0.57 | -1.74 (-6.39˗2.91) | 0.46 | 0.56 (-0.57˗1.68) | 0.33 | -1.09 (-5.55˗3.37) | 0.63 | 0.03 (-1.12˗1.19) | 0.95 | -0.41 (-2.03˗1.20) | 0.62 | -0.10 (-2.61˗2.42) | 0.94 | 0.79 (-1.85˗3.43) | 0.56 |
| G/A vs G/G | -1.56 (-15.15˗12.04) | 0.82 | -5.72 (-51.15˗39.72) | 0.81 | -1.60 (-6.60˗3.40) | 0.53 | 0.79 (-0.42˗2.00) | 0.20 | -0.73 (-5.51˗4.06) | 0.77 | 0.07 (-1.18˗1.31) | 0.92 | -0.40 (-2.14˗1.34) | 0.65 | -0.27 (-2.97˗2.43) | 0.84 | 0.69 (-2.15˗3.53) | 0.63 |
| A/A vs G/G | -20.65 (-86.93˗45.62) | 0.54 | -107.91 (-329.41˗113.60) | 0.34 | -5.27 (-29.63˗19.08) | 0.67 | -1.80 (-7.70˗4.09) | 0.55 | -6.72 (-30.05˗16.62) | 0.57 | -0.35 (-6.36˗5.67) | 0.91 | -0.94 (-9.34˗7.45) | 0.83 | 1.94 (-11.08˗14.97) | 0.77 | 2.79 (-10.91˗16.49) | 0.69 |
| **rs7675998** | 2.06 (-4.76˗8.88) | 0.55 | 4.52 (-18.32˗27.35) | 0.70 | 1.49 (-1.02˗3.99) | 0.25 | 0.19 (-0.42˗0.80) | 0.54 | 1.41 (-0.99˗3.81) | 0.25 | 0.29 (-0.33˗0.91) | 0.35 | 0.86 (-0.02˗1.74) | 0.06 | 0.77 (-0.61˗2.14) | 0.27 | 1.04 (-0.40˗2.48) | 0.16 |
| G/A vs G/G | -0.55 (-9.29˗8.2) | 0.90 | 2.79 (-26.5˗32.08) | 0.85 | 0.72 (-2.49˗3.94) | 0.66 | 0.31 (-0.47˗1.09) | 0.44 | 0.73 (-2.34˗3.81) | 0.64 | -0.04 (-0.84˗0.75) | 0.92 | 0.46 (-0.66˗1.58) | 0.42 | 0.00 (-1.75˗1.75) | 1.00 | 0.12 (-1.71˗1.95) | 0.90 |
| A/A vs G/G | 9.35 (-8.15˗26.84) | 0.30 | 12.48 (-46.12˗71.09) | 0.68 | 4.50 (-1.93˗10.93) | 0.17 | 0.15 (-1.41˗1.70) | 0.86 | 4.19 (-1.96˗10.35) | 0.18 | 1.25 (-0.34˗2.83) | 0.12 | 2.54 (0.26˗4.81) | **0.03** | 3.13 (-0.41˗6.68) | 0.08 | 4.00 (0.29˗7.72) | **0.04** |
| **rs9420907** | -0.48 (-8.31˗7.35) | 0.90 | -10.26 (-36.49˗15.98) | 0.44 | -1.57 (-4.41˗1.27) | 0.28 | -0.36 (-1.04˗0.33) | 0.31 | -2.24 (-4.96˗0.48) | 0.11 | -0.09 (-0.8˗0.62) | 0.81 | -0.30 (-1.30˗0.71) | 0.56 | 0.12 (-1.43˗1.67) | 0.88 | -0.42 (-2.03˗1.19) | 0.61 |
| A/C vs C/C | -3.88 (-13.51˗5.75) | 0.43 | -18.32 (-50.61˗13.96) | 0.27 | -0.32 (-3.81˗3.17) | 0.86 | -0.43 (-1.27˗0.41) | 0.32 | -1.23 (-4.58˗2.12) | 0.47 | 0.18 (-0.69˗1.06) | 0.68 | 0.05 (-1.19˗1.29) | 0.94 | 0.68 (-1.23˗2.59) | 0.48 | 0.24 (-1.74˗2.23) | 0.81 |
| A/A vs C/C | 9.17 (-13.73˗32.07) | 0.43 | 3.53 (-73.27˗80.32) | 0.93 | -6.88 (-15.19˗1.44) | 0.11 | -0.49 (-2.49˗1.50) | 0.63 | -7.49 (-15.46˗0.48) | 0.07 | -0.99 (-3.07˗1.09) | 0.35 | -1.60 (-4.51˗1.31) | 0.28 | -1.38 (-5.88˗3.11) | 0.55 | -2.78 (-7.45˗1.90) | 0.24 |
| **rs6772228** | 26.31 (4.52˗48.09) | **0.02** | 76.24 (3.25˗149.23) | **0.04** | 2.18 (-5.85˗10.20) | 0.60 | -0.71 (-2.66˗1.24) | 0.48 | 1.62 (-6.07˗9.31) | 0.68 | 0.35 (-1.64˗2.33) | 0.73 | 1.09 (-1.69˗3.86) | 0.44 | 2.23 (-2.08˗6.54) | 0.31 | 0.77 (-3.75˗5.29) | 0.74 |
| T/A vs T/T | 26.31 (4.52˗48.09) | **0.02** | 76.24 (3.25˗149.23) | **0.04** | 2.18 (-5.85˗10.20) | 0.60 | -0.71 (-2.66˗1.24) | 0.48 | 1.62 (-6.07˗9.31) | 0.68 | 0.35 (-1.64˗2.33) | 0.73 | 1.09 (-1.69˗3.86) | 0.44 | 2.23 (-2.08˗6.54) | 0.31 | 0.77 (-3.75˗5.29) | 0.74 |
| **rs10936599** | -0.93 (-8.29˗6.42) | 0.80 | -0.17 (-24.71˗24.38) | 0.99 | -0.97 (-3.64˗1.70) | 0.48 | -0.02 (-0.67˗0.64) | 0.96 | -1.08 (-3.63˗1.48) | 0.41 | -0.01 (-0.68˗0.66) | 0.98 | -0.17 (-1.11˗0.78) | 0.73 | -0.64 (-2.10˗0.83) | 0.39 | -0.54 (-2.05˗0.96) | 0.48 |
| C/T vs C/C | -0.68 (-9.67˗8.31) | 0.88 | 1.28 (-28.71˗31.26) | 0.93 | -2.04 (-5.30˗1.21) | 0.22 | 0.18 (-0.61˗0.98) | 0.65 | -2.05 (-5.17˗1.07) | 0.20 | -0.11 (-0.92˗0.71) | 0.79 | -0.47 (-1.63˗0.70) | 0.43 | -0.99 (-2.78˗0.81) | 0.28 | -1.06 (-2.90˗0.79) | 0.26 |
| T/T vs C/C | -2.61 (-23.46˗18.25) | 0.81 | -4.47 (-74.04˗65.10) | 0.90 | 1.16 (-6.40˗8.71) | 0.76 | -0.60 (-2.44˗1.25) | 0.53 | 0.63 (-6.61˗7.87) | 0.86 | 0.27 (-1.62˗2.16) | 0.78 | 0.50 (-2.16˗3.16) | 0.71 | -0.31 (-4.41˗3.79) | 0.88 | 0.34 (-3.87˗4.56) | 0.87 |
| **rs2736100** | 3.86 (-2.36˗10.07) | 0.22 | 7.54 (-13.36˗28.44) | 0.48 | -1.01 (-3.29˗1.26) | 0.38 | -0.21 (-0.77˗0.35) | 0.47 | -0.70 (-2.90˗1.50) | 0.53 | -0.13 (-0.70˗0.43) | 0.65 | 0.34 (-0.46˗1.13) | 0.41 | 0.72 (-0.52˗1.96) | 0.26 | 0.18 (-1.13˗1.49) | 0.79 |
| C/A vs C/C | 3.47 (-6.46˗13.39) | 0.49 | 10.54 (-22.83˗43.90) | 0.54 | -0.39 (-4.03˗3.25) | 0.83 | -0.26 (-1.15˗0.64) | 0.58 | 0.34 (-3.17˗3.85) | 0.85 | -0.63 (-1.53˗0.28) | 0.17 | 0.09 (-1.19˗1.37) | 0.89 | 0.86 (-1.13˗2.86) | 0.40 | -0.45 (-2.55˗1.66) | 0.68 |
| A/A vs C/C | 7.81 (-4.78˗20.39) | 0.22 | 14.35 (-27.97˗56.66) | 0.51 | -2.18 (-6.80˗2.43) | 0.35 | -0.40 (-1.53˗0.73) | 0.49 | -1.65 (-6.11˗2.80) | 0.47 | -0.15 (-1.29˗1.00) | 0.80 | 0.73 (-0.89˗2.34) | 0.38 | 1.41 (-1.10˗3.91) | 0.27 | 0.50 (-2.14˗3.14) | 0.71 |
| **rs755017** | 4.98 (-6.20˗16.16) | 0.38 | 16.68 (-20.72˗54.08) | 0.38 | 2.74 (-1.36˗6.83) | 0.19 | -0.55 (-1.55˗0.44) | 0.27 | 2.27 (-1.66˗6.20) | 0.26 | 1.00 (-0.01˗2.01) | 0.05 | 0.43 (-0.99˗1.86) | 0.55 | 0.06 (-2.17˗2.29) | 0.96 | 0.26 (-2.08˗2.59) | 0.83 |
| A/G vs A/A | 5.07 (-6.40˗16.54) | 0.39 | 17.64 (-20.73˗56.01) | 0.37 | 2.12 (-2.08˗6.31) | 0.32 | -0.53 (-1.55˗0.49) | 0.31 | 1.67 (-2.35˗5.70) | 0.42 | 0.91 (-0.12˗1.95) | 0.08 | 0.32 (-1.14˗1.79) | 0.66 | 0.37 (-1.92˗2.65) | 0.75 | 0.18 (-2.21˗2.58) | 0.88 |
| G/G vs A/A | 6.69 (-87.05˗100.43) | 0.89 | -1.52 (-315.14˗312.11) | 0.99 | 27.89 (-6.41˗62.18) | 0.11 | -2.12 (-10.44˗6.21) | 0.62 | 26.10 (-6.79˗58.99) | 0.12 | 5.16 (-3.29˗13.62) | 0.23 | 4.65 (-7.2˗16.51) | 0.44 | -10.69 (-29.11˗7.73) | 0.26 | 3.20 (-16.09˗22.49) | 0.75 |
| **rs8105767** | 4.53 (-2.02˗11.08) | 0.18 | 9.51 (-12.45˗31.47) | 0.40 | -0.88 (-3.29˗1.53) | 0.48 | -0.29 (-0.88˗0.29) | 0.32 | -0.79 (-3.09˗1.52) | 0.50 | -0.19 (-0.79˗0.41) | 0.54 | -0.35 (-1.2˗0.49) | 0.41 | -0.98 (-2.29˗0.34) | 0.14 | -0.43 (-1.81˗0.94) | 0.54 |
| A/G vs A/A | 5.44 (-3.44˗14.32) | 0.23 | 5.85 (-23.91˗35.62) | 0.70 | -2.12 (-5.38˗1.15) | 0.20 | -0.09 (-0.88˗0.70) | 0.82 | -2.21 (-5.34˗0.91) | 0.17 | -0.49 (-1.3˗0.31) | 0.23 | -0.65 (-1.79˗0.5) | 0.27 | -2.39 (-4.16˗-0.61) | **0.01** | -1.57 (-3.43˗0.30) | 0.10 |
| G/G vs A/A | 7.77 (-7.83˗23.38) | 0.20 | 24.18 (-28.14˗76.51) | 0.37 | 0.00 (-5.74˗5.73) | 1.00 | -0.88 (-2.27˗0.51) | 0.22 | 0.44 (-5.05˗5.93) | 0.87 | 0.06 (-1.37˗1.50) | 0.93 | -0.30 (-2.3˗1.71) | 0.77 | 0.00 (-3.10˗3.11) | 1.00 | 0.71 (-2.55˗3.97) | 0.67 |
| **rs412658** | 4.09 (-2.10˗10.28) | 0.20 | 6.72 (-14.02˗27.47) | 0.53 | 0.05 (-2.21˗2.31) | 0.96 | -0.21 (-0.76˗0.35) | 0.46 | -0.06 (-2.24˗2.12) | 0.96 | 0.02 (-0.55˗0.59) | 0.95 | -0.18 (-0.98˗0.62) | 0.65 | 0.02 (-1.22˗1.27) | 0.97 | -0.19 (-1.49˗1.11) | 0.78 |
| C/T vs C/C | 2.76 (-6.30˗11.83) | 0.55 | 7.32 (-23.06˗37.69) | 0.64 | -1.11 (-4.42˗2.2) | 0.51 | 0.09 (-0.71˗0.90) | 0.82 | -0.95 (-4.14˗2.24) | 0.56 | -0.15 (-0.98˗0.68) | 0.72 | -0.40 (-1.57˗0.77) | 0.51 | -1.34 (-3.16˗0.48) | 0.15 | -1.43 (-3.34˗0.48) | 0.14 |
| T/T vs C/C | 9.19 (-4.17˗22.56) | 0.18 | 13.00 (-31.79˗57.78) | 0.57 | 0.98 (-3.90˗5.86) | 0.69 | -0.64 (-1.83˗0.55) | 0.29 | 0.56 (-4.14˗5.26) | 0.82 | 0.17 (-1.06˗1.39) | 0.79 | -0.21 (-1.92˗1.51) | 0.81 | 1.04 (-1.61˗3.70) | 0.44 | 0.53 (-2.27˗3.32) | 0.71 |

Linear regressions of the SNPs on the sperm parameters. The additive and codominant inheritance models were used, with the more common allele as reference. Analyses are adjusted for age and smoking status.
