# Supplementary material for: Telomere Length and Male Fertility
Source: Int J Mol Sci. 2021 Apr 12;22(8):3959. doi: 10.3390/ijms22083959 (PMC8069448; doi:10.3390/ijms22083959)
Supplement: Supplementary file 1 [file ijms-22-03959-s001.zip › Supplementary table/Supplementary Table AIII.docx]

**Supplementary Table AIII:** Study population description

|  |  |
| --- | --- |
| **Number of patients** | 599 |
| **Smoker no/yes** | 374/191 |
| **Sperm parameters** | **Median (Q_1_-Q_3_)** |
| Age (years) | 36 (30-40) |
| Concentration (mln/mL) | 39 (14-82) |
| Total number (mln) | 107 (38-234) |
| Progressive motility (%) | 49 (35-60) |
| Non-progressive motility (%) | 0 (0-2) |
| Total motility (%) | 52 (38-61) |
| Normal morphology (%) | 6 (4-10) |
| Normal acrosome (%) | 9 (6-13) |
| Normal head (%) | 22 (14-29) |
| Normal flagellum (%) | 37 (30-42) |

Q_1_: first quartile; Q_3_: third quartile. Number of missing data: smoke N = 2; concentration N = 30; total number N = 32; progressive motility N = 30; non-progressive motility N = 31; total motility N = 30; normal morphology N = 42; normal acrosome N = 66; normal head N = 68; normal flagellum N = 68.
