# Supplementary material for: Telomere Length and Male Fertility
Source: Int J Mol Sci. 2021 Apr 12;22(8):3959. doi: 10.3390/ijms22083959 (PMC8069448; doi:10.3390/ijms22083959)
Supplement: Supplementary file 1 [file ijms-22-03959-s001.zip › Supplementary table/Supplementary Table AIV.docx]

**Supplementary Table AIV.** SNPs reported to be associated with telomere length by previously published GWAS.

| **Gene** | **SNP** | **Chromosome** | **Position** | **EA^a^** | **OA^a^** | **EAF^a^** | **β (SE)^a^** | **Base pairs^b^** | **Discovery P_value_** | **Discovery study** |
| --- | --- | --- | --- | --- | --- | --- | --- | --- | --- | --- |
| *ACYP2* | rs11125529 | 2 | 54,248,729 | A | C | 0.11 | 0.065 (0.012) | 78.0 | 8.00×10^−10^ | Codd et al. (37) |
| *PXK* | rs6772228 | 3 | 58,390,292 | T | A | 0.96 | 0.041 (0.014) | 49.2 | 3.91×10^−10^ | Haycock et al. (39) |
| *TERC* | rs10936599 | 3 | 169,774,313 | C | T | 0.76 | 0.100 (0.011) | 120.0 | 3.00×10^−31^ | Codd et al. (37) |
| *NAF1* | rs7675998 | 4 | 163,086,668 | G | A | 0.76 | 0.048 (0.012) | 57.6 | 4.35×10^−16^ | Codd et al. (37) |
| *TERT* | rs2736100 | 5 | 1,286,401 | G | T | 0.50 | 0.085 (0.013) | 102.0 | 4.38×10^−19^ | Codd et al. (37) |
| *OBFC1* | rs9420907 | 10 | 103,916,707 | C | A | 0.13 | 0.142 (0.014) | 170.4 | 7.00×10^−11^ | Codd et al. (37) |
| *CTC1* | rs3027234 | 17 | 8,232,774 | C | T | 0.78 | 0.103 (0.012) | 123.6 | 2.00×10^−8^ | Mangino et al. (38) |
| *ZNF208* | rs8105767 | 19 | 22,032,639 | G | A | 0.28 | 0.064 (0.011) | 76.8 | 1.11×10^−9^ | Codd et al. (37) |
| *ZNF676* | rs412658 | 19 | 22,359,440 | T | C | 0.35 | 0.086 (0.010) | 103.2 | 1.00×10^-8^ | Mangino et al. (38) |
| *DHX35* | rs6028466 | 20 | 39,500,359 | A | G | 0.07 | 0.058 (0.013) | 69.6 | 2.57×10^−8^ | Haycock et al. (39) |
| *ZBTB46* | rs755017 | 20 | 62,421,622 | G | A | 0.12 | 0.019 (0.013) | 22.8 | 6.71×10^−9^ | Codd et al. (37) |

^a^ EA: effect allele is the allele associated with long leukocyte telomere length in previously published studies. OA: other allele; EAF: effect allele frequency in Caucasian population; β (SE): estimate of telomere length variation for each copy of EA as reported in Haycock. ^b^ Base pairs: estimate in base pairs of telomere length variation for each copy of EA, using a formula (bp = beta x 1200) extrapolated from Codd et al. (2013)
